# Subgrouping patients with sciatica in primary care for matched care pathways: development of a subgrouping algorithm

**DOI:** 10.1186/s12891-019-2686-x

**Published:** 2019-07-04

**Authors:** Kika Konstantinou, Kate M. Dunn, Danielle van der Windt, Reuben Ogollah, Vinay Jasani, Nadine E. Foster, the SCOPiC study team, Majid Artus, Elaine M. Hay, Martyn Lewis, Jonathan C. Hill, Christian Mallen

**Affiliations:** 1 Primary Care Centre Versus Arthritis, Research Institute for Primary Care & Health Sciences, Keele University, Staffordshire, ST5 5BG UK; 2 Haywood Hospital, Midlands Partnership NHS Foundation Trust, Stoke-on-Trent, Staffordshire, ST6 7AG UK; 3 Keele Clinical Trials Unit, Keele University, Staffordshire, ST5 5BG UK; 4 Department of Spine Surgery, University Hospital North Midlands, Royal Stoke University Hospital, Newcastle Rd, Stoke-on-Trent, ST4 6QG UK; 5 Present address: Nottingham Clinical Trials Unit, School of Medicine, University of Nottingham, Nottingham, NG7 2UH UK

**Keywords:** Sciatica, Algorithm, Stratification, Leg pain, Care pathway, Referral

## Abstract

**Background:**

Sciatica is a painful condition managed by a stepped care approach for most patients. Currently, there are no decision-making tools to guide matching care pathways for patients with sciatica without evidence of serious pathology, early in their presentation. This study sought to develop an algorithm to subgroup primary care patients with sciatica, for initial decision-making for matched care pathways, including fast-track referral to investigations and specialist spinal opinion.

**Methods:**

This was an analysis of existing data from a UK NHS cohort study of patients consulting in primary care with sciatica (*n* = 429). Factors potentially associated with referral to specialist services, were identified from the literature and clinical opinion. Percentage of patients fast-tracked to specialists, sensitivity, specificity, positive and negative predictive values for identifying this subgroup, were calculated.

**Results:**

The algorithm allocates patients to 1 of 3 groups, combining information about four clinical characteristics, and risk of poor prognosis (low, medium or high risk) in terms of pain-related persistent disability. Patients at low risk of poor prognosis, irrespective of clinical characteristics, are allocated to group 1. Patients at medium risk of poor prognosis who have all four clinical characteristics, and patients at high risk of poor prognosis with any three of the clinical characteristics, are allocated to group 3. The remainder are allocated to group 2. Sensitivity, specificity and positive predictive value of the algorithm for patient allocation to fast-track group 3, were 51, 73 and 22% respectively.

**Conclusion:**

We developed an algorithm to support clinical decisions regarding early referral for primary care patients with sciatica. Limitations of this study include the low positive predictive value and use of data from one cohort only. On-going research is investigating whether the use of this algorithm and the linked care pathways, leads to faster resolution of sciatica symptoms.

**Electronic supplementary material:**

The online version of this article (10.1186/s12891-019-2686-x) contains supplementary material, which is available to authorized users.

## Background

Sciatica (radicular pain) has significant impact on patients, healthcare and societal costs [1]. The annual impact on the United Kingdom (UK) economy is £268 million in direct medical costs, and £1.9 billion in indirect costs, based on a Dutch study which indicated that the societal cost of sciatica represents 13% of all back pain related costs (inflated from 1998 figures) [2]. Many patients with sciatica do well over time and are treated successfully with conservative interventions, with injections and/or surgery reserved for patients with persistent, disabling pain [3].

In the absence of suspected serious pathology or profound neurological deficits, current usual management of sciatica in the UK National Health Service (NHS) follows a ‘stepped’ care approach, with an initial period of ‘low level’ treatment for most patients, including advice, reassurance and analgesic medication, and for those not improving, onward referrals are initiated to services such as physiotherapy or specialist spinal services in intermediate and secondary care settings [4]. Up to 30% of patients with severe sciatic pain continue having significant symptoms at 1 year follow-up [5]. However, a direct referral to spinal specialist services for all sciatica patients at the point of first consultation in primary care is not a cost-effective care model [6], and it is unlikely that this is needed for most patients, except when sinister pathology is suspected. In the UK, most primary care patients with sciatica, who may eventually require a specialist opinion, are referred to NHS spinal specialist services at the interface (intermediate) level, between primary and secondary care services. Interface spinal services are predominately staffed by non-medically qualified clinicians (for example physiotherapists) with advanced training and expertise in spinal care and direct referral access to surgical and injection services [4]. The challenge for clinicians (such as general practitioners (GPs)) managing sciatica in primary care is how to identify, early in the presentation, patients who are likely to only need and do well with conservative management in primary care, and patients who may need early, fast-track referral to spinal specialist opinion.

For non-specific low back pain (LBP), research has shown that a model of stratified care that does not over-treat those with a good chance of improvement but identifies those likely to need more intensive treatment, has led to superior clinical and cost effectiveness compared to non-stratified care [7, 8]. This approach in LBP utilises a brief self-completed screening tool (the Keele STarT Back tool) [9] which captures eight modifiable physical and psychological prognostic indicators. The STarT Back tool has 9 items, 4 are physical constructs, with 2 questions capturing back pain/leg pain related disability, and 5 are psychological constructs. A score of 3 or less indicates the patient is at low risk of future persistent back pain-related disability, a score of 4 or more of the 5 psychological items indicates the patient is at high risk, any other score identifies patients as at medium risk of persistent disability [9]. By estimating future risk of persistent back pain-related disability, the STarT Back tool supports early clinical decision-making about conservative treatments (such as GP care and physiotherapy management) [7, 9]. Although neither the STarT Back trial [7] nor the subsequent implementation study (IMPaCT Back) [8] excluded patients with sciatica, the STarT Back tool and matched treatments were not specifically developed for sciatica. Sciatica patients have more severe pain and take longer to recover [1]. Currently there is no screening tool or algorithm available to support clinical decision-making in directing patients with sciatica to matched care pathways, when they first consult with sciatica symptoms which are not suspicious of sinister pathology. This is explained, at least in part, by the currently limited number of prognostic factors that have been shown to be independently associated with outcome in patients with sciatica [10–12]. This paucity of evidence for prognostic factors, with only pain and disability severity being consistently associated with eventually having spinal surgery (which is taken as proxy of poor outcome for natural course and conservative management) [11, 13], impedes development of prognostic models that can guide treatment decision-making [11], yet it is not feasible or necessary to refer all patients with high levels of pain and disability to spinal specialist or surgical services. However, early referral to investigations and more intensive treatments may improve outcomes and quicker recovery for some sciatica patients consulting in primary care, instead of waiting the outcome of initial conservative management before referring on when there is no improvement.

The aim of this research was to develop an algorithm with which to support clinical decision-making about subgrouping patients with sciatica consulting in primary care for matched care pathways.

## Methods

### Study population: ATLAS cohort

In order to identify the subgroup of sciatica patients likely to need fast-track specialist referral, we used data from the ATLAS study. ATLAS is a prospective, treatment cohort of primary care patients with sciatica [12, 14, 15] that investigated their overall prognosis, 1 year after consultation in UK general practice. Full details and results of ATLAS have been described elsewhere [12, 15]; here we give brief details of the ATLAS study’s procedures. Patients consulting their GP with back and leg pain, including sciatic symptoms, were invited to participate in the ATLAS study. They were assessed by experienced musculoskeletal physiotherapists for purposes of eligibility and diagnosis (referred leg pain or sciatica), and were treated according to clinical need and best current practice. Patients were included irrespective of pain severity, so there was a comprehensive representation from very mild pain to severe. Patients with suspected serious pathologies were excluded from the study. The assessing physiotherapists were asked to state their level of confidence (as a percentage 0 to 100%) in their clinical diagnosis for each participant. All participants without contra-indications to magnetic resonance imaging (MRI) had a scan for research purposes. The MRI did not inform the initial clinical diagnosis of sciatica. Most patients in the ATLAS study received physiotherapy treatment, with a small number of patients being referred for a specialist opinion for consideration of further management options such as spinal injections or surgery, if their symptoms did not respond to conservative management and taking into account their MRI findings [14]. For the development of the algorithm we included ATLAS participants diagnosed with sciatica with diagnostic confidence of 70% and over (as reported by the assessors) [16, 17]. The outcome definition was referral to NHS spinal specialist services (yes/no).

### Selection of potential predictors of referral for specialist opinion

Potential predictors of referral to specialist services were selected from self-report and clinical examination. The choice of factors was based on previous literature [10–12, 18–22], clinical opinion and experience, and availability in the ATLAS dataset. The selected factors were categorised into 5 domains: impact of condition, pain levels/symptoms, psychological perceptions, symptom behaviour and presentation, and clinical examination findings. We were broad in our approach of including potential factors due to the paucity of evidence for strong prognostic factors. The 5 domains and the factors selected, the rationale for each, and details of how they were measured, are summarised in Additional file 1.

### Identification of factors associated with fast-track referral to spinal specialist services in patients with sciatica

#### Step 1: descriptive analysis

Descriptive statistics (means and standard deviations for continuous variables and frequency counts and percentages for categorical variables) on key demographic and clinical characteristics, and risk of poor prognosis (STarT Back tool (see Additional file 2)), were calculated according to referral/no referral to specialist services.

#### Step 2a: statistical analysis: factors associated with referral to spinal specialist services

Logistic regression was used to determine the association between each factor and the outcome of referral to spinal specialist services. Factors were entered in blocks according to each domain. First, univariable analysis was performed to assess the association between each factor in each domain and the outcome. Tests of multicollinearity were performed for all the variables within each domain, first by pairwise correlations (Pearson’s correlation for numerical scales, Spearman’s correlation for ordinal categorical measures, and point-biserial correlation for binary covariates), with one variable dropped if correlation coefficient > 0.7 and then by variance inflation factor (VIF > 5 considered as evidence of collinearity). Second, multivariable analysis comprising all variables within each domain (excluding correlated variables if identified) was performed separately in each domain to identify independent factors within each domain. Third, all the independent factors from each domain were entered in a final multivariable model to identify factors independently associated (*p* < 0.05) with the outcome of referral to specialist services.

#### Step 2b: clinical relevance of factors associated with referral to spinal specialist services

The factors derived from the multivariable regression models as being most strongly associated with referral to specialist services, within each domain and overall, were further discussed with the study’s multidisciplinary clinical and research advisory group (three epidemiologists, four trialists, two statisticians, one spinal surgeon, five spinal physiotherapy specialists, one rheumatologist, one pain specialist, five GPs). The final list of factors were chosen for their clinical relevance and face validity for referral to specialist services. Performance of the final model was measured by its discriminative ability and calibration. The ability of the model to discriminate between referred and non-referred patients was summarised using the area under the receiver operating characteristic curve (AUC), with 95% CIs. In the absence of an external validation cohort, internal validation was performed with the bootstrap procedure as described by Steyerberg et al. [23] using 500 replications to estimate optimism in performance. The calibration slope with 95% CI was calculated to examine whether the predicted probabilities agreed with the observed probabilities.

#### Step 2c: identifying patients for fast-track referral to spinal specialist services: algorithm design

In this step of the iterative process, we investigated a number of possibilities in terms of combinations of factors from the clinical assessment and information on risk of poor prognosis, using the STarT Back Tool score, for identifying which patients with sciatica to refer or ‘fast-tracked’ to spinal specialist services. For each combination of factors, we considered the implications for sensitivity in terms of identifying observed referrals, and feasibility and practicality in relation to use in clinical practice. For all scenarios, sensitivity/specificity, positive/negative predictive values, and percentage of sample fast-tracked, were calculated. This information was used in constructing the algorithm to guide clinical decision-making in primary care about referrals for patients with sciatica. The results were discussed with the clinical and research advisory group, and the most optimal and feasible option was chosen.

## Results

### Step 1: descriptive characteristics

Of the 609 study participants, 429 were diagnosed with sciatica by the musculoskeletal physiotherapists with at least 70% diagnostic certainty and formed the sample used for algorithm development. Of these, 57 (13.3%) were referred, at some point during 12 months follow-up, to NHS spinal specialist services for further assessment and treatment. Table 1 shows baseline characteristics of participants referred / not referred to specialist services. Referred patients had higher levels of leg pain and disability compared to those not referred, and higher proportions had neurological deficits such as weakness and sensory deficits in the affected leg. In terms of risk of poor prognosis (STarT Back Tool), only one out of the 57 patients referred to spinal specialists was in the low risk subgroup on the STarT Back Tool. Patients in the high risk STarT Back subgroup had higher probability of being in the group referred to specialists (54.5% vs 37.8%), and those in the medium risk STarT Back subgroup were proportionally almost equally divided between those referred and not referred to spinal specialists (43.6% vs 49.2%).

**Table 1:** Baseline characteristics for patients according to referral to specialist spinal services. Figures are frequencies (percentage) unless otherwise specified

	Referred to spinal specialist *N* = 57^a^	Not referred *N* = 372
Age (years) (mean, SD)	53.7 (15.0)	49.7 (13.8)
Gender (F)	30 (52.7)	230 (62.0)
Leg pain usual intensity (mean, SD)	8.0 (1.8)	6.7 (2.3)
Leg pain worse than back pain	35 (61.4)	206 (55.4)
Disability (RMDQ) (mean, SD)	15.4 (4.6)	12.6 (5.8)
Duration of leg pain:		
less than 6 weeks	11 (20.4)	172 (47.8)
between 6 weeks to 3 months	18 (33.3)	73 (20.3)
over 3 months	25 (46.3)	115 (32.0)
STarT Back prognostic risk subgroup:		
Low	1 (1.8)	47 (13.1)
Medium	24 (43.6)	177 (49.2)
High	30 (54.5)	136 (37.8)
Neurological examination findings:		
Myotomal weakness	20 (36.0)	82 (22.0)
Reflex deficits	16 (28.0)	90 (24.2)
Sensory deficits	37 (65.0)	182 (49.0)
Neural tension tests:		
Positive	40 (70.2)	272 (73.1)

^a^There was a small proportion of missing data for some baseline characteristics so the numbers may not add up to 57 exactly

### Step 2a: factors associated with referral to spinal specialist services

Table 2 presents the results of the univariable and multivariable analysis for each domain of factors, and the final model including all four independent factors. Three factors were independently significantly associated with referral to spinal specialist services: impact of back or leg pain on ability to do job or work around the house, leg pain intensity and sensory deficit in the painful leg, found on clinical assessment. The fourth factor, pain self-efficacy, was not statistically significant in the overall model. The final model’s apparent performance (based on the AUC (95% CI)) for the original sample was 0.695 (0.622, 0.768). The mean of performance in the 500 bootstrapped samples was 0.713 (0.710, 0.716), with an expected optimism of 0.018, thus the internally validated (optimism adjusted) performance was 0.678 (0.674, 0.681). The calibration slope (95% CI) for this model was 1.0 (0.57, 1.43) showing that the model was well-calibrated, albeit with uncertainty reflected by the wide confidence interval.

**Table 2:** Baseline characteristics of sciatica patients by referral status (to spinal specialist services) and unadjusted and adjusted odds ratios of association with referral

	Not referred (*n* = 372)	Referred (*n* = 57)	Unadjusted^a^ OR (95% CI)	Adjusted^b^ OR (95% CI)
Block 1				
Impaired performance at work or cannot do jobs around the house: Yes^*^, n (%)	200 (53.8)	43 (75.4)	**2.64 (1.40, 4.99)**	**2.17 (1.13, 4.17)**
Block 2				
Intensity of usual back pain in the last 2 weeks, mean (SD)	7.0 (2.2)	7.4 (2.1)	1.10 (0.96, 1.26)	
Intensity of usual leg pain in the last 2 weeks, mean (SD)	6.7 (2.3)	8.0 (1.8)	**1.32 (1.14, 1.53)**	
Intensity of current back pain, mean (SD)	5.4 (2.7)	6.3 (2.6)	**1.13 (1.02, 1.27)**	
Intensity of current leg pain, mean (SD)	5.5 (2.9)	6.9 (2.5)	**1.20 (1.07, 1.34)**	**1.17 (1.05–1.31)**
Sciatica Bothersomeness (0–6): total score, median (IQR)				
Leg pain	5 (3, 6)	6 (4, 6)	**1.58 (1.22, 2.05)**	
Numbness or tingling in leg, foot or groin (Paraesthesia)	4 (2, 5)	5 (3, 6)	**1.25 (1.06, 1.48)**	
Weakness in leg or foot	3 (1, 4)	4 (2, 5)	**1.20 (1.04, 1.38)**	
Back or leg pain while sitting	4 (3, 5)	5 (3, 6)	**1.34 (1.09, 1.65)**	
Sciatica bothersomeness composite score (0–24): mean (SD)	14.6 (5.1)	17.6 (4.5)	**1.13 (1.06, 1.21)**	
Block 3				
Pain self-efficacy (0–60), mean (SD)	34.1 (14.5)	28.0 (15.0)	**0.97 (0.95, 0.99)**	–
Illness perception (identity), median (IQR)	6 (5, 7)	6 (5, 7)	1.23 (0.96, 1.57)	
Block 4				
What is worse: Leg pain is worse, n (%)	206 (55.4)	35 (61.4)	1.28 (0.72, 2.27)	
Tingling/numbness: Yes, n (%)	241 (64.8)	40 (70.2)	1.28 (0.70, 2.34)	
Cough/sneeze positive: Yes, n (%)	97 (26.1)	20 (35.1)	1.53 (0.85, 2.77)	
Block 5				
Abnormal myotomal strength: Yes, n (%)	82 (22.0)	20 (35.1)	**1.91 (1.05, 3.47)**	
Reflex, n (%)				
Normal	282 (75.8)	41 (71.9)		
Slightly reduced	28 (7.5)	2 (3.5)	0.49 (0.11, 2.14)	
Absent	46 (12.4)	11 (19.3)	1.64 (0.79, 3.43)	
Significantly reduced	16 (4.3)	3 (5.3)	1.29 (0.36, 4.62)	
Sensation (Pin Prick), n (%)				
Normal	190 (51.1)	20 (35.1)		
Reduced sensation	142 (38.2)	26 (45.6)	1.74 (0.93, 3.24)	1.59 (0.84–3.00)
Loss of sensation	40 (10.8)	11 (19.3)	**2.61 (1.16, 5.88)**	**2.41 (1.05–5.53)**
Neural test: any positive: Yes, n (%)	272 (73.1)	40 (70.2)	0.87 (0.47, 1.60)	

^*^Evidence of interference with ability to do work/home activities if > 6 (NRS 0–10) on single question on work interference or ‘yes’ response on the RMDQ item

^a^ Univariable association between each variable and referral to secondary care, variables with *p* < 0.05 are bolded

^b^ Multivariable model was fitted in two stages: (1) within each block: block 1 only had one variable; block 2 had 9 variables, however, variables within this block were all highly correlated with each other and could not be included in the same multivariable model, current leg pain was chosen on clinical grounds to be the only variable progressed to multivariable model; block 3 had two variables but one was not significant when adjusted within the block and was dropped; block 4 had 3 variables, none were significant when adjusted for each other within the block; block 5 had 4 variables, only one was significant when adjusted within the block; (2) overall adjusted model comprising all variables significant in the adjusted models within blocks: the following variables were entered in the initial overall multivariable model: impaired performance at work or cannot do jobs around the house, intensity of current leg pain, pain self-efficacy, and sensation. Pain self-efficacy was not significant and was dropped from the final model

### Step 2b: clinical relevance of factors associated with referral to spinal specialist services

The clinical and research advisory group discussed and agreed the final list of factors thought to be most relevant in the decision to refer to spinal specialists. The following four factors were chosen: effect of low back and/or leg pain on ability to do one’s job or ability to do jobs around the house (Numerical Rating Scale (NRS) 0–10, cut-off point > 6, for those not in work ‘yes’ response on the RMDQ item about ability to do jobs around the house), current leg pain intensity (NRS 0–10, cut-off point > 6), sensory deficits in a dermatomal distribution recorded during the clinical examination (yes/no), and pain below the knee (yes/no). The first three factors were derived from the statistical analyses. The binary cut-off points (NRS > 6) for impact and pain intensity were agreed by the advisory group as reasonable thresholds for pain and functional limitations and with face validity when considering an early referral to specialists. Presence of pain below the knee is considered the best proxy indicator of leg pain due to nerve root involvement [24], the advisory group considered this to be important to include, in combination with the other factors for making referral decisions, therefore this factor was subsequently added to the list.

### Step 2c: identifying patients for fast-track referral to spinal specialist services: algorithm design

In this step, three scenarios were investigated. The first two scenarios investigated only characteristics (factors) from the clinical examination associated with fast-track referral to spinal specialists, using two cut-off points. The third scenario combined prognostic information (from the STarT Back Tool) with the clinical characteristics, and investigated combinations of clinical characteristics and prognostic subgroups (medium, high risk); Table 3 presents the results of this analysis. In the first scenario (only considering clinical characteristics: 3 of the 4 present), sensitivity was 68%, but it would fast-track 43% of the whole sample to spinal specialists. In the second scenario (all 4 clinical characteristics present), only 14% of the sample would be fast-tracked, but sensitivity was lower (32%), so a significant number of patients needing a spinal specialist referral would be missed. The third scenario combined information about the clinical characteristics and prognostic subgroup. Patients with a STarT Back score indicating low risk of persistent back pain-related disability, irrespective of clinical characteristics present, were not considered in the analysis for identifying those for fast-tracking to specialist, as only one such patient in the ATLAS cohort was observed as referred to specialists over a period of 12 months follow-up; thus we considered that very few patients with such good prognosis would need fast-tracking to spinal specialists. The combination considered optimal by the research and clinical advisory group for fast-tracking sciatica patients to spinal specialist services was a classification of high risk on the STarT Back Tool and at least 3 of the 4 clinical characteristics present, or a classification of medium risk on the STarT Back Tool and all 4 clinical characteristics present. This resulted in sensitivity of 51% and specificity of 73% with approximately one third of the whole sample (30%) being identified for ‘fast-track’ referral. The positive predictive value (PPV) value was 22%, indicating that 22% of all the fast-track referral patients in this sample, would be appropriately referred for specialist opinion. This third scenario was chosen as the most clinically appropriate for identifying patients for fast-track referral, based on sensitivity value and feasibility in terms of ‘fast-track’ referral numbers to spinal specialist services.

**Table 3:** Identification of patients for ‘fast-track’ referrals to spinal specialist services. Three scenarios investigated

	Sensitivity (95% CI^a^)	Specificity (95% CI)	Positive predictive value (95% CI)	Negative predictive value (95% CI)	% of total sample referred
Use of only clinical characteristics (total of 4^b^):					
- Patients with 3 out of 4 clinical characteristics	68% (55–80)	60% (55–65)	21% (15–27)	93% (88–96)	43% (186/429)
- Patients with all 4 clinical characteristics	32% (20–45)	89% (85–92)	30% (19–44)	89% (86–92)	14% (59/429)
Use of clinical characteristics combined with STarT Back prognostic risk score (medium, high)^c^					
- patients at ‘high’ risk on STarT Back, with 3 or more clinical characteristics	51% (37–64)	73% (68–78)	22% (16–31)	91% (87–94)	30% (129/429)
OR					
- patients at ‘medium’ risk on STarT Back, with all 4 clinical characteristics					

^a^CI; Confidence Intervals

^b^Current leg pain > 6 (NRS 0–10), pain below knee, interference with ability to do work/home activities > 6 (NRS 0–10) or ‘yes’ response on the RMDQ item, sensory deficit in painful leg with pin/prick testing

^c^Patients with a low risk STarT Back score were excluded from this analysis, as from the ATLAS cohort, only one patient at low risk of poor outcome was referred to spinal specialist services

### Full algorithm

**Fig. 1:**
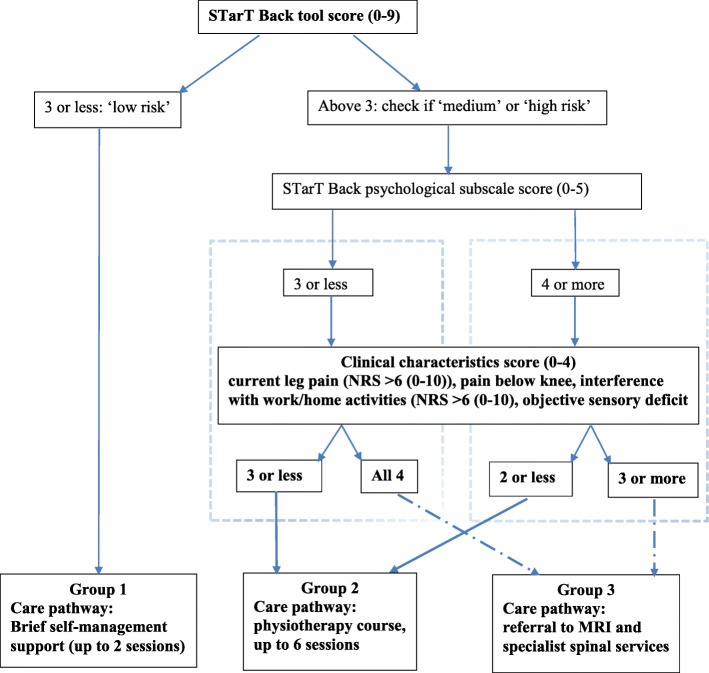
Stratification algorithm for allocating patients to groups and matched care pathways

Figure 1 shows the suggested algorithm which allocates sciatica patients to 1 of 3 groups, and summarises the suggested matched care pathways. The allocation of patients to group 1 was based only on prognostic risk information from the STarT Back Tool (as only one participant classified at low risk was referred) (see Table 1). The allocation of patients to groups 2 and 3 was informed by the analysis described in previous sections for identifying patients needing a referral to spinal specialist services. Table 4 presents key characteristics for the three groups defined by the subgrouping algorithm when applied in the ATLAS cohort.

**Table 4:** Key baseline characteristics for patients according to the groups formed by applying the stratification algorithm in the ATLAS cohort dataset. Figures are frequencies (proportions) unless otherwise specified

	Group 1 (*n* = 52) STarT Back low risk	Group 2 (*n* = 266) Combination of up to 3 clinical characteristics and STarT Back medium risk or up to 2 clinical characteristics and STarT Back high risk	Group 3 (*n* = 111) Combination of at least 3 clinical characteristics and STarT Back high risk or 4 clinical characteristics and STarT Back medium risk
Characteristics			
Age (years) (mean, (SD^a^))	52.5 (13.44)	49.5 (13.6)	51.0 (15.0)
Gender (F)	55.7 (29)	61.6 (164)	60.4 (67)
Low back pain intensity (mean, (SD))	2.5 (2.4)	5.5 (2.4)	7.0 (2.3)
Leg pain intensity (mean, (SD))	3.5 (2.6)	5.2 (2.7)	8.0 (1.9)
Leg pain worse than back pain	65.4 (34)	52.6 (140)	60.3 (67)
Disability (RMDQ^b^) (mean, (SD))	5.7 (3.4)	12.6 (5.0)	17.3 (4.4)
Pain below knee	66.0 (33)	70.2 (179)	94.5 (104)
Duration of leg pain:			
less than 6 weeks	52.0 (26)	45.7 (116)	37.3 (41)
between 6 weeks to 3 months	24.0 (12)	21.7 (55)	21.8 (24)
over 3 months	24.0 (12)	32.7 (83)	41.0 (45)
HADs^c^ depression score (mean, (SD))	2.8 (2.4)	6.1 (3.6)	8.6 (4.3)
PSEQ^d^ (mean, (SD))	48.9 (8.8)	34.5 (13.0)	22.8 (13.0)
Neurological examination findings:			
Myotomal weakness (any)	13.5 (7)	21.8 (58)	33.3 (37)
Reflex deficit (any)	15.4 (8)	23.3 (62)	32.4 (36)
Sensory deficit (any)	44.2 (23)	41.0 (109)	78.4 (87)
Neural tension tests:			
any positive	63.5 (33)	74.9 (199)	72.1 (80)

^a^SD: standard deviation

^b^Roland Morris Disability Questionnaire

^c^Hospital Anxiety and Depression scale

^d^Pain self-efficacy Questionnaire

Analyses were performed using Stata 14 (StataCorp. 2015. Stata Statistical Software: Release 14. College Station, TX: StataCorp LP).

## Discussion

We have developed an algorithm for supporting clinical decisions regarding onward referral at the point of initial consultation, and allocation to matched care pathways, for patients consulting in primary care with sciatica. The algorithm combines information about patients’ risk of persistent back and leg pain related disability and four items from the clinical assessment. It allocates patients to one of three groups for matched care pathways: for group 1 this comprises of brief self-management support (up to 2 sessions), for group 2 a course of up to 6 sessions of physiotherapist-led care, and for group 3 a ‘fast-track’ referral to MRI and spinal specialist services.

Currently, prognostic research in sciatica patients shows that only one or two factors (pain, disability or impact of the condition/symptoms) are consistently associated with persistent disability or proceeding to surgery (when surgery is an appropriate and desirable management option) [11–13, 25], but not all sciatica patients with severe leg pain will have a poor outcome and will not proceed to have surgery. This limits the development of useful prognostic models to inform stratified care and guide clinical decision-making about early referral to spinal specialists. In the absence of models purely based on probability of poor outcome for this condition, the next reasonable step is to consider investigating and combining information on prognosis with factors associated with referral to specialists. It is possible that referral to specialists for investigations and treatments early in the presentation of symptoms in primary care, may improve outcomes for some sciatica patients however, this will need to be investigated in clinical trials. To our knowledge, this is the first subgrouping algorithm developed to guide clinical management at first consultation in primary care for patients with sciatica.

It was important that the factors/characteristics used, would be acceptable to clinicians in terms of directing referrals to NHS spinal specialist services immediately after consultation in primary care, given the possible impact of early ‘fast-tracking’ of patients on the healthcare system. In choosing the ‘optimal’ combination of characteristics and cut-off points, we considered the trade-offs between sensitivity (correctly identifying patients who need a referral to spinal specialists) and feasibility, as it would be impractical to refer the majority of patients. And the input of our research and clinical advisory group was unanimous and fundamental in these decisions, indicating high clinical face validity. We acknowledge as a limitation that the clinical advisory group did not include patients. The model’s predictive performance is moderate (C-statistic: 0.70). The positive predictive value (PPV) of the algorithm for specialist referral is 22%, with sensitivity of 51%. It is clear that based on the proposed algorithm, a number of patients will be referred early for tests (MRI) and spinal specialist assessment, who may not have needed such referral. It is fully acknowledged that these values are low, however, in the absence of a reference standard or evidence-based guidance to underpin referral decisions at first consultation (in the absence of suspected sinister pathology), we consider this as a first step towards stratified care for this population. In the UK, these patients are referred to primary/secondary care interface (intermediate) clinics where they are further evaluated, appropriate management options are discussed with the patient, and referrals from that intermediate setting are streamlined to surgical, injections or pain management settings, in secondary care.

Further potential limitations relate to the factors, and their cut off points, we included in the algorithm development. We chose the list of factors based on published data and clinical opinion, and that were available in the ATLAS cohort study dataset. We included all factors that could, in principle, be associated with referral to spinal specialist services and could also be easily collected/recorded during a routine consultation with a GP or physiotherapist. We were practical about the need for brevity and simplicity for use of such an algorithm in busy clinical practice. Although the algorithm presented in Fig. 1 appears cumbersome, if its use for directing patients’ care at first consultation was to lead to better outcomes compared to the current model of stepped care, the algorithm can be fashioned in a ‘calculator’ form, embedded in the current electronic systems used by GPs, in the same way such systems are used for calculating cardiovascular risk for example.

Although we tested the final model using recommended internal validation statistics, we acknowledge the limitation of using data from only one cohort. Further testing in other similar populations will need to provide evidence on external validity, however, we are not aware that a similar primary care cohort is available. At this stage, we do not recommend the algorithm is used in clinical practice, as clinical trials are necessary first to assess the effects of the algorithm on patients’ outcomes. To that effect, we are currently testing the clinical and cost-effectiveness of using this subgrouping (stratification) algorithm with matched care pathways, compared to non-stratified care, in a randomised controlled trial (SCOPiC; ISRCTN75449581) [26].

## Additional files

Additional file 1: Factors considered for their potential association with referral to spinal specialist services. (DOCX 24 kb)

Additional file 2: STarT Back Tool. (DOC 40 kb)

## Data Availability

The datasets used and analysed during the current study are available from the corresponding author on reasonable request.

## Abbreviations

AUC: Area Under Curve
CI: Confidence Interval
GP: General Practitioner
LBP: Low Back Pain
MRI: Magnetic Resonance Imaging
NHS: National Health System
NRS: Numerical Rating Scale
PPV: Positive Predictive Value
UK: United Kingdom
VIF: Variance Inflation Factor
