# Supplementary material for: Subgrouping patients with sciatica in primary care for matched care pathways: development of a subgrouping algorithm
Source: BMC Musculoskelet Disord. 2019 Jul 4;20:313. doi: 10.1186/s12891-019-2686-x (PMC6611047; doi:10.1186/s12891-019-2686-x)
Supplement: Supplementary file 1 — Factors considered for their potential association with referral to spinal specialist services. (DOCX 24 kb) [file 12891_2019_2686_MOESM1_ESM.docx]

Additional file 1. Factors considered for their potential association with referral to spinal specialist services

|  | **Domain** | **Rationale** | **Measure** |
| --- | --- | --- | --- |
| 1 | Impact of condition | The impact of sciatic symptoms on patient’s ability to do work or other necessary activities of daily living is taken into account by clinicians in relation to referral decisions. High disability levels are associated with proceeding to surgery in patients with sciatica due to disc prolapse (Peul et al 2008). | Single question on work interference: ‘*On average, to what extent has your back or leg pain affected your performance at work since your back or leg pain started?*’ Rated on a 0-10 scale where 0 is ‘not at all’ and 10 is ‘the pain is so bad that I am unable to do my job’  For those patients not at work: single question on back and leg pain related disability from the Roland and Morris Disability Questionnaire (RMDQ) (Patric et al 1995): ‘*Because of my back or leg problem, I am not doing any of the jobs that I usually do around the house*.’ Response: yes/no  For the stratification algorithm, these two variables were combined into one binary variable (with a 1 for a score of 7 or more on the Numerical Rating Scale (NRS) 0-10 or a “Yes” response to RMDQ question, and a 0 otherwise) to identify patients with impaired ability to do their work or jobs around the house (for those not in work). |
| 2 | Pain levels: low back pain, leg pain  Sciatica bothersomeness | Pain intensity is taken into account by clinicians in relation to referral decisions. Literature indicates leg pain intensity as a factor associated with having spinal surgery in disc-related radicular symptoms (Verwoerd et al 2013, Peul et al 2008).  Specific sciatica symptoms impact. Limited evidence on its use as part of referral decision, therefore the research team considered investigating. | Intensity of usual back pain in the last 2 weeks (NRS 0-10).  Intensity of usual leg pain in the last 2 weeks.  Intensity of back pain at present time.  Intensity of leg pain at present time.  (Dunn et al 2010).  Sciatica Bothersomeness Index (SBI) (Grovle et al 2010): patient’s rating on four symptoms (leg pain, numbness or tingling in leg, foot or groin, weakness in leg or foot, and back or leg pain while sitting) on a 0-6 point scale, according to how bothersome they were in the past week, where 0 is ‘not bothersome’ and 6 is ‘extremely bothersome’. SBI composite score (0-24) is derived from the 4 symptoms. |
| 3 | Psychological perceptions | Ability to cope and have control over symptoms is more strongly associated with outcome in LBP patients, compared to other characteristics such as depression (Foster et al 2010).  Similarly, the ‘identity’ variable is associated with poorer outcomes in LBP (Foster et al 2010). | Pain self-efficacy: Measured with the Pain Self-Efficacy Questionnaire (PSEQ); with summated scores of 10 items from 0 to 60; higher scores reflect stronger self-efficacy beliefs (Nicholas 2007)  Identity: Symptom attribution to the condition (Moss-Morris et al 2002) from a list of 7 potential symptoms: back pain, leg pain, unable to sit comfortably, fatigue, stiff joints, sleep difficulties, loss of strength. The score is the sum of symptoms experienced. |
| 4 | Symptom presentation and behaviour | Symptom behaviour may contribute to diagnostic decision making and potentially to referral decision making. | Pins and needles or numbness in leg(s) as reported by the patient.  Leg pain increased by coughing/laughing/straining.  Worse pain, either in low back or leg.  Pain below knee**^b^** |
| 5 | Clinical examination, findings from neurological assessment | Neurological examination is part of routine spinal assessment. Assessment findings contribute to diagnosis and may contribute to referral decision making. | Neurological examination variables:  -Myotomal strength**^c^**; defined as normal (5 on Oxford scale)/abnormal (0,1,2,3, or 4 on Oxford scale)  -Reflex (tendon); defined as normal, slightly reduced, significantly reduced/absent  -Sensation**^d^**; (in leg(s)) approximating a dermatomal distribution, defined as normal/reduced/loss of sensation  -Neural tension test findings; defined as abnormal if any neural tension test is abnormal (i.e. straight leg raise, femoral stretch, slump) |

**^a^**Timeline and Personal control are measured on a Likert scale; Strongly disagree - Disagree - Neither agree or disagree - Agree - Strongly agree. For the purposes of the analysis it was dichotomised ((agree *(agree, strongly agree)* versus disagree *(strongly disagree, disagree, neither agree or disagree))*.

**^b^** The variable ‘below knee pain’ was not included in the regression analysis but was subsequently incorporated in the algorithm development, at the advice of the clinical advisory group.

**^c^** Muscle strength tested according to the Oxford scale where; 0. No movement, 1. Flicker of movement, 2. Through full range actively with gravity counterbalanced, 3. Through full range actively against gravity, 4. Through full range actively against some resistance, 5. Through full range actively against strong resistance.

**^d^** Sensation was tested with a pin (neurotip).

References

Peul WC; Brand R; Thomeer RTWM; Koes BW. Improving prediction of "inevitable" surgery during non-surgical treatment of sciatica. Pain 2008; 138(3):571–76

Verwoerd AJH, Luijsterburg PAJ, Lin CWC et al. Systematic review of prognostic factors predicting outcome in non-surgically treated patients with sciatica. Eur J Pain 2013; 17: 1126-37.

Dunn KM, Jordan KP, Croft PR. Recall of medication use, self-care activities and pain intensity: a comparison of daily diaries and self-report questionnaires among low back pain patients. Prim Health Care Res Dev. 2010;11:93–102.

Patrick DL, Deyo RA, Atlas SJ et al. Assessing health-related quality of life in patients with sciatica. Spine 1995;20: 1899-1908.

Nicholas MK. The pain self-efficacy questionnaire: taking pain into account. Eur J Pain. 2007;11:153–63

Moss-Morris R, Weinman J, Petrie KJ, et al. The Revised Illness Perceptions Questionnaire (IPQ-R). Psychol Health. 2002;17(1):1–16.

Foster NE, Thomas E, Bishop A et al. Distinctiveness of psychological obstacles to recovery n low back pain patients in primary care. Pain 2010; 148: 398–406.
