# Supplementary material for: Subgrouping patients with sciatica in primary care for matched care pathways: development of a subgrouping algorithm
Source: BMC Musculoskelet Disord. 2019 Jul 4;20:313. doi: 10.1186/s12891-019-2686-x (PMC6611047; doi:10.1186/s12891-019-2686-x)
Supplement: Supplementary file 2 — STarT Back Tool. (DOC 40 kb) [file 12891_2019_2686_MOESM2_ESM.doc]

# Additional file 2. STarT Back Tool

# The Keele STarT Back Screening Tool

Thinking about the **last 2 weeks** tick your response to the following questions:

|  |  | **Disagree** | **Agree** |
| --- | --- | --- | --- |
|  |  | 0 | 1 |
| 1 | My back pain has **spread down my leg(s)** at some time in the last 2 weeks | □ | □ |
| 2 | I have had pain in the **shoulder** or **neck** at some time in the last 2 weeks | □ | □ |
| 3 | I have only **walked short distances** because of my back or leg pain | □ | □ |
| 4 | In the last 2 weeks, I have **dressed more slowly** than usual because of back or leg pain | □ | □ |
| 5 | It’s not really safe for a person with a condition like mine to be physically active | □ | □ |
| 6 | **Worrying thoughts** have been going through my mind a lot of the time | □ | □ |
| 7 | I feel that **my back/leg pain is terrible** and **it’s never going to get any better** | □ | □ |
| 8 | In general I have **not enjoyed** all the things I used to enjoy | □ | □ |

9. Overall, how **bothersome** has your back or leg pain been in the **last 2 weeks**?

| Not at all | Slightly | Moderately | Very much | Extremely |
| --- | --- | --- | --- | --- |
| □ | □ | □ | □ | □ |
| 0 | 0 | 0 | 1 | 1 |

**Total score (all 9): __________________ Sub Score (Q5-9):______________**

© Keele University 01/08/07

Funded by Arthritis Research UK

# The STarT Back Tool Scoring System

**Total score**

3 or less

4 or more

**Sub score Q5-9**

3 or less

4 or more

Low risk

Medium risk

High risk

© Keele University 01/08/07

Funded by Arthritis Research UK
